# Is Lifelong Physical Activity a Determinant of Lung Transplantation Outcomes?

**DOI:** 10.3390/jcm15072738

**Published:** 2026-04-04

**Authors:** Natalia Muklewicz, Marta Gallas, Bartosz Sławomir Żegleń, Katarzyna Barbara Grzegorczyk, Marta Żołnowska, Anna Katarzyna Góra, Karolina Lipka, Krzysztof Chmura, Aleksandra Gradek, Rafał Nojek, Marta Piotrowska, Marcin Sawczuk, Filip Szydzik, Julia Anita Tarnowska, Jacek Wojarski, Wojciech Karolak, Sławomir Żegleń

**Affiliations:** 1 University Clinical Centre in Gdańsk, 80-952 Gdańsk, Poland; nmuklewicz@uck.gda.pl (N.M.); agradek@uck.gda.pl (A.G.); marpiotrowska@szpitalepomorskie.eu (M.P.); j.tarnowska@gumed.edu.pl (J.A.T.); 2 Department of Cardiac Surgery, University Clinical Centre in Gdańsk, 80-214 Gdańsk, Poland; 3 Department of Preventive Medicine & Education, Medical University of Gdańsk, 80-211 Gdańsk, Poland; 4 Independent Team of Physiotherapists, University Clinical Centre in Gdańsk, 80-952 Gdańsk, Poland; 5 Department of Applied Computer Science, AGH University of Science and Technology, 30-059 Kraków, Poland; dornier1997@gmail.com; 6 Department of Endocrinology and Internal Diseases, Medical University of Gdańsk, 80-214 Gdańsk, Poland; 7 Department of Cardiac Surgery, University Clinical Centre in Gdańsk, Medical University of Gdańsk, 80-214 Gdańsk, Polandwkarolak@uck.gda.pl (W.K.); 8 Department of Pulmonology, Medical University of Gdańsk, 80-214 Gdańsk, Poland

**Keywords:** long-term physical activity, lung transplantation, lung transplantation outcomes

## Abstract

**Background:** Habitual physical activity (PhA) may contribute to overall physiological reserve, yet its relevance for lung transplantation (LTx) remains unclear, as existing studies almost exclusively explore short-term exercise interventions. This study evaluates whether life-course PhA before LTx influences postoperative outcomes: functional capacity measured by 6 min walk test (6MWT), chronic lung allograft dysfunction (CLAD), and length of hospital stay (LOS). **Methods:** In this retrospective study, ninety-seven LTx recipients completed the Historical Adulthood Physical Activity Questionnaire (HAPAQ), assessing PhA from age 20 to transplantation. All participants were assessed within the same time frame, with varying time intervals since transplantation. Patients were classified into no/recreational/intensive sport groups based on regular participation. Statistical analyses examined sport group differences in 6MWT, CLAD, and LOS and interactions with sex, pulmonary disease type, and secondary pulmonary hypertension (PH). **Results:** Lifelong PhA did not significantly differentiate quantitative 6MWT, CLAD, or LOS, and no interaction effects were observed. A modest trend was noted in patients without secondary PH, among whom intensive PhA corresponded to more frequent within-norm qualitative 6MWT results. Outcomes were comparable between single- and double-lung (DLTx) recipients, although a moderate effect between sports groups suggested a possible DLTx compensatory advantage. **Conclusions:** This study provides the very first life-course assessment of PhA in LTx recipients, highlighting the value of HAPAQ for estimating pre-transplant physiological reserve. Despite postoperative outcomes being largely independent of lifelong PhA, and recovery appearing multifactorial, habitual PhA should not be overlooked in developing individualized prehabilitation strategies in transplant medicine.

## 1. Introduction

Lung transplantation (LTx) is a recognized, life-saving treatment for carefully selected patients with end-stage pulmonary diseases, including interstitial lung disease, chronic obstructive pulmonary disease (COPD), emphysema, cystic fibrosis, bronchiectasis, or pulmonary hypertension (PH) [1,2]. Despite major surgical and pharmacological improvements, graft failure, rejection, and infections remain key concerns after LTx and lead to poorer post-transplant outcomes [3,4].

Evidence suggests that maintaining an active lifestyle supports immune function, which may positively impact health and disease progression, particularly in older adults. Regular exercise over the life course has been associated with a reduced risk of cardiovascular diseases, cancer, bacterial and viral infections, and other chronic inflammatory disorders, suggesting improved immune competence [5]. Physical exercise may further modulate immunological responses, which could be relevant to graft rejection, although these mechanisms have not been specifically established in the context of lung transplantation and remain to be fully elucidated [6].

Physical activity (PhA) is generally known to benefit overall health, and growing evidence indicates that it exerts beneficial effects both before and after LTx [7]. However, while most studies focus on short-term exercise interventions, especially related to postoperative or strictly preoperative period, a research gap remains regarding the influence of habitual PhA initiated in early adulthood and continued throughout the adult life course, on functional capacity, and related recovery or quality of life (QoL) after transplantation.

The present study aims to evaluate the association between lifelong PhA behavior before LTx and post-transplant outcomes, including exercise capacity measured by the six-minute walk test (6MWT), chronic lung allograft dysfunction (CLAD)/broncholitis obliterates syndrome (BOS) stage, and length of hospital stay after LTx (LOS).

## 2. Materials and Methods

### 2.1. Study Population

Between April and July 2025, 101 patients under follow-up at the Outpatient Clinic of the Department of Lung Transplantation, University Clinical Centre in Gdańsk, Poland, were invited to participate. All patients underwent LTx between July 2018 and March 2025. As the lung transplantation program at our center has been active since 2018, all consecutive eligible patients attending follow-up visits during the study period were enrolled, resulting in variable time since transplantation at assessment. During 1-day outpatient visit, participants completed a questionnaire assessing their life-course PhA before surgery.

Exclusion criteria were incorrectly completed questionnaires (3 patients) and being underage at the time of transplantation (1 patient). Ultimately, 97 participants were included in the final analyses (28 women, 29%; 69 men, 71%). The mean age was 57 years. Single-lung transplantation (SLTx) was performed in 17 patients (17.5%) and double-lung transplantation (DLTx) in 80 (82.5%), including 3 combined transplant procedures (2 lung-heart and 1 lung-kidney).

The study was approved by the Bioethics Committee for Scientific Research at the Medical University of Gdańsk (approval no. NKBBN/677/2020). Informed consent was obtained from all participants before their inclusion in the study.

### 2.2. PhA Assessment

The Historical Adulthood Physical Activity Questionnaire (HAPAQ) [8] was used to assess habitual, life-course PhA from age 20 to the time of the study. Despite the open access, the full version of the questionnaire and permission to translate it to Polish were obtained from the Medical Research Council (MRC) Epidemiology Unit, University of Cambridge. The instrument captures activity undertaken at home, at work, during commuting, and during leisure time through repeated close-ended questions on the type, frequency, and duration of activity across predefined life periods. The first section covers the past 15 years in 5-year intervals, while the second assesses adulthood in 10-year intervals (20–29, 30–39, etc.). Both sections consist of the same questions and address household characteristics, domestic chores, commuting habits (walking/cycling), occupational activity (sedentary, standing, manual) and leisure-time exercise, distinguishing between recreational and vigorous (resulting in breathlessness or sweating) activity, with items specifying duration and type of sport performed [8].

Before completion, the questionnaire was explained individually to each patient and life periods were reconstructed using personal milestones (e.g., graduation, marriage, childbirth) to aid recall. Our study team was available throughout the process to clarify any uncertainties. Because the duration of instructed daily activities (e.g., gardening, housework, do-it-yourself tasks) could not be reliably quantified when reported imprecisely (e.g., ‘all my life’, ‘all the time’), these items were excluded from the analysis to ensure consistency and minimize potential misclassification of exposure.

### 2.3. Sport Group Classification

Patients were assigned to 1 of 3 sport groups based on the consistent participation (≥5 years threshold) in active commuting or regular recreational or vigorous PhA before LTx. The ≥5-year threshold was based on the structure of the HAPAQ, which distinguishes 5-year and 10-year assessment intervals. As age at transplantation varied across participants, a 5-year period was considered a consistent indicator of sustained, regular PhA across participants.

Group 1 (no sport): no or <5 years of active commuting or recreational PhA in any period of life before LTx.

Group 2 (recreational sport): >5 years of active commuting or recreational PhA or <5 years of vigorous PhA in any period of life before LTx.

Group 3 (intensive sport): ≥5 years of vigorous PhA in any period of life before LTx.

### 2.4. Clinical Data Collection

Clinical data were extracted from electronic medical records, including LOS, underlying pulmonary disease, presence of secondary PH, and type of transplantation. LOS was calculated from the LTx date to the date of first discharge home.

For selected analyses, underlying pulmonary diseases were categorized as obstructive (e.g., COPD, alpha-1 antitrypsin deficiency) or restrictive (e.g., idiopathic pulmonary fibrosis, pneumoconiosis, hypersensitivity pneumonitis, other interstitial lung diseases), with additional stratification based on the presence of secondary PH. Due to distinct pathophysiology, patients with primary PH and those who underwent heart–lung transplantation were excluded from these subgroup analyses.

Functional capacity after LTx was evaluated using the 6MWT, and graft function (CLAD) was assessed by determining the BOS stage for each patient. All clinical outcomes were subsequently analyzed in relation to the assigned sport group.

### 2.5. 6MWT

Functional capacity was assessed with the standardized 6MWT after LTx [9]. It was based on the most recent available assessment at the time of the follow-up. The test was performed in a flat, 30 m corridor. Vital signs, oxygen saturation, and Borg dyspnea scores were recorded before and after the test. Saturation and pulse were monitored every minute during the walk with finger or ear sensor. Patients were encouraged and walked at a self-selected pace with standing rests permitted. The test was terminated for sustained desaturation <80% or sitting down. Distance walked was compared with reference values calculated using validated Enright-Sherrill equations, which adjust for age, sex, height, and weight, and both expected values and the lower limit of normal were derived individually using established norm [9,10,11,12].

Formulas for reference 6MWT distance [11,12]:

**Men:**

Upper limit = (7.57 × height [cm]) − (5.02 × age) − (1.76 × body weight [kg]) − 309
Lower limit = upper limit − 153 m

**Women:**

Upper limit = (2.11 × height [cm]) − (2.29 × body weight [kg]) − (5.78 × age) + 667
Lower limit = upper limit − 139 m

### 2.6. CLAD Assesment

BOS, a phenotype of CLAD, was defined as ≥20% decrease in FEV_1_ from the post-transplant baseline persisting ≥3 weeks due to persistent small airway obstruction, with other causes ruled out [13,14]. Baseline FEV_1_ was calculated as the mean of the 2 highest post-transplant values ≥3 weeks apart without bronchodilator use [13,15]. The most recent FEV_1_ was compared with the baseline, and BOS was graded as follows: BOS 0: FEV_1_ > 90% of baseline, BOS 0-p: 81–90%, BOS 1: 66–80%, BOS 2: 51–65%, BOS 3: ≤50% [15]. BOS grading was based on the most recent spirometric assessments at follow-up and was not standardized to a fixed post-transplant time point. For BOS analyses, patients had to be ≥3 months post-transplant to allow sufficient time for BOS development and baseline spirometry assessment [14]. Accordingly, 7 patients were excluded.

### 2.7. Statistical Analysis

Statistical analyses were performed using IBM SPSS Statistic 29. Basic descriptive statistics were calculated alongside the Shapiro–Wilk test to assess the normality of the distributions. A two-way between-subjects analysis of variance (ANOVA) was conducted, and a Student’s *t*-test for independent samples for dependent variables measured on a quantitative scale for which the mean and standard deviation were reported. For dependent variables measured on an ordinal scale, the Mann–Whitney U test and Kruskal–Wallis test were applied, with the median and interquartile range reported. Additionally, chi-square tests of independence were used for dependent variables measured on a nominal scale, with frequencies and percentages presented. A significance level of α = 0.05 was adopted.

## 3. Results

### 3.1. 6MWT—Quantitative Approach

Certain factors within the patient group differentiated the quantitative outcomes of the 6MWT and whether these factors interacted with one another. To this end, a series of ANOVA was conducted. In each model, the dependent variable was the 6MWT result, with one of the independent variables being sport group (no sport vs. recreational sport vs. intensive sport), and the other independent variables, which varied across the models, included sex (female vs. male), pulmonary disease (obstructive vs. restrictive), and the presence of secondary PH (no vs. yes). The results are presented in Table 1.

The analysis revealed that neither the main effects of sport groups, sex, pulmonary disease or secondary PH, nor the interactions between sport groups and sex, sport groups and pulmonary disease, or sport groups and secondary PH reached statistical significance. Effect sizes were small across all models. These findings indicate that 6MWT results did not differ across patient groups defined these variables and the relationship between PhA and 6MWT was not moderated by them.

### 3.2. 6MWT—Qualitative Approach

Subsequently, it was examined whether there were factors differentiating the qualitative outcomes of the 6MWT within the studied patient group. To this end, a chi-square test of independence was used to compare patients differentiated by PhA in terms of the qualitative results of the 6MWT. The analysis was conducted both overall and stratified by sex, obstructive/restrictive pulmonary disease, and secondary PH. The results are presented in Table 2.

The analysis revealed a statistically significant difference between the compared groups among patients without secondary PH. Within this subgroup, individuals who reported engaging in the intensive sport achieved within-norm test results significantly more often than those reporting no PhA. Moreover, patients without secondary PH who reported no sport achieved above-norm results significantly more frequently than those engaging in recreational sport. It is also noteworthy that the observed effect size was moderate (0.30 < Vc < 0.50).

### 3.3. BOS Stage

The next step of the analysis was to examine potential factors differentiating the BOS stage among the studied patient group. The Kruskal–Wallis test was employed to compare BOS severity across sport groups. The analysis was conducted for the entire group as well as within subgroups stratified by sex, obstructive/restrictive disease, and the presence of secondary PH. The results are presented in Table 3.

The analysis did not reveal any statistically significant differences between the compared groups in terms of BOS stage. That indicated that patients differentiated by sport levels, both overall and within predefined subgroups, did not differ in BOS severity.

### 3.4. LOS Analysis

In each model, the dependent variable was the LOS with one independent variable being the sport group. The remaining independent variables, which varied across the analyzed models, including sex, pulmonary disease, and the presence of secondary PH. The results are presented in Table 4.

Patient groups differing in PhA level (main effect of sport groups), by sex (main effect of sex), by obstructive/restrictive disease profile (main effect of obstructive/restrictive disease), and by the presence of PH (main effect of secondary PH) did not differ in terms of LOS.

### 3.5. Comparison of Physically Active SLTx Recipients with Physically Inactive DLTx Recipients in Terms of 6MWT, BOS Stage and LOS

Subsequently, it was examined whether physically active patients (recreational and/or intensive sport), who had undergone SLTx, achieved better outcomes than physically inactive patients (reporting no sport) after DLTx. The results are presented in Table 5.

The analysis did not reveal any statistically significant differences between the compared patient sport group in terms of the studied post-transplant outcomes. This indicates that physically active SLTx recipients did not differ from physically inactive DLTx recipients with respect to the 6MWT (both quantitative and qualitative), BOS stage, and LOS. However, a moderate effect size was observed for the qualitative 6MWT outcome (0.30 < Vc < 0.50, which may suggest a potential trend that did not reach statistical significance, likely related to the limited sample size. Given the exploratory nature of this comparison, the presence of multiple interacting factors, and a small sample size, these findings should be interpreted with caution.

## 4. Discussion

This study evaluated the association between lifelong habitual PhA before LTx and post-transplant results, that is, functional capacity (6MWT), graft function (CLAD), and hospitalization time (LOS). To our knowledge, this is the first study to investigate life-course PhA as a determinant of outcomes in solid organ transplantation. While interest in prehabilitation before transplantation is growing, the current existing research examines short-term exercise interventions rather than long-term exercise patterns. However, lifelong PhA habits may contribute to physiological resilience and influence post-transplant recovery potential.

Prior studies primarily have used short-term, self-report tools such as the PASE [16], IPAQ [17,18], or TOAQ [19], typically assessing activity within the preceding week. A longer observation has been reported only in isolated studies, for example, with the use of the MLTPAQ (12 months) [19]. Longitudinal assessments remain rare, although the HAPAQ [8] offers a validated approach for retrospective lifetime evaluation. Integrating such tools into pre-transplant assessments and qualification process for transplantation may provide better insight into patients’ PhA history and their potential physiological reserve and could support the development of more individualized rehabilitation strategies.

It is well established that regular PhA improves overall health, physical fitness, and survival by reducing the risk of cardiovascular disease, diabetes, hypertension, or depression, but in transplant populations, PhA may provide additional benefits—improved QoL, reduced immunosuppressive burden, and better graft preservation [20,21,22,23]. Frailty and reduced exercise capacity among transplant candidates strongly predict poorer postoperative outcomes, including mortality [24]. It was observed that frailty, which is reported in up to 70% of LTx candidates, was associated with longer hospitalizations, higher readmission rates, physical and psychological decline, and increased mortality [25]. Moreover, mortality appears more closely linked to pre-transplant functional status than to post-transplant changes in PhA [16]. Structured exercise programs proved to preserve physical fitness during the waiting period [26,27], highlighting the need for standardized prehabilitation guidelines.

### 4.1. Functional Capacity (6MWT)

The 6MWT is a widely accepted measure of exercise capacity and a strong predictor of survival among LTx patients, regardless of their underlying lung disease [28]. In their study, Larger and colleagues [29] emphasized the importance of identifying, understanding, and modifying sedentary lifestyle as a part of pre-transplant care.

Contrary to expectations, our results showed no significant differences in quantitative 6MWT performance among LTx candidates with different habitual PhA levels. Neither sex, lung disease type, nor the presence of secondary PH influenced this relationship. Importantly, these findings indicate that self-reported PhA alone may not adequately reflect post-transplant functional capacity, which is likely determined by multiple factors, including psychological and clinical ones.

Several studies observed mixed effects of structured prehabilitation programs on 6MWT in LTx candidates. Bourgeois et al. [30] reported stable rather than improved performance after a 12-week virtual prehabilitation program, whereas Florian et al. [31] and Kenn et al. [32] found significant gains of 56–72 m following intensive training. Similar results were observed by Gloeckl and colleagues [27], who demonstrated improvements with both continuous and interval training, though without significant differences between these protocols. In contrast, among lung and heart transplant candidates, Wallen et al. reported no difference in 6MWT, VO_2_ peak, or QoL in training participants versus control group, despite within-group improvements [33].

In other transplant populations, the FRAILMar trial [34] found no significant 6MWT change in kidney transplant candidates after 8-week prehabilitation, while Ma et al. [35] demonstrated benefits from a 12-week remotely supervised program. Likewise, Foresteri et al. [36] and Ben-Gal et al. [37] observed improvements in heart transplant candidates with deterioration in physical condition in non-training patients. Among liver transplant candidates, it was reported that each 100 m increase in 6MWT was associated with a 52% reduction in immortality risk [38], and a threshold of 401.8m was identified as predictive of clinical decompensation [39]. Several studies [40,41,42] confirmed that 12-week structured or home-based prehabilitation improves 6MWT and VO_2_ peak and reduces frailty progression. Collectively, these data indicate that prehabilitation may enhance exercise capacity, but effects depend on disease severity, exercise supervision, and intervention design.

Our assumption that patients who led a more favorable lifestyle in the context of PhA would have greater physiological reserves, potentially translating into better results of the LTx procedure, was not confirmed in our study. Perhaps it is the severity of the disease that is so devastating and decisive, regardless of the initial stage. Furthermore, the aspect of an individualized therapeutic window remains important. Currently, the same qualification criteria are applied irrespective of the initial patient status. It raises the question of whether more individualized assessment of organ impairment in each patient could be considered. 

### 4.2. Secondary PH

In our cohort, patients without secondary PH, who reported intensive sport participation, were more likely to reach 6MWT results within the normal range, suggesting potential benefits for patients with less advanced cardiopulmonary limitations. Larger et al. [29] found that LTx candidates typically perform about 52% below the 6MWT reference values, with exercise capacity being the strongest determinant of daily activity. Our results suggest that maintaining regular PhA may help offset that decline. Interestingly, some inactive patients without PH achieved above-normal 6MWT scores, representing a paradox that may reflect self-assessment bias, challenges in PhA classification, compensatory physiological mechanisms, other unmeasured factors, or may be due to chance.

Recent evidence supports the benefit of respiratory and exercise training even in severe PH and right heart failure. Mereles et al. [43] reported significant improvements in 6MWT and oxygen uptake, while Munawar et al. [44] observed a modest 18 m gain in the pre-transplant period, but also stable exercise capacity despite progressive disease. Taken together, these results reinforce that functional performance in the transplant candidates reflects an interaction of clinical, physiological, and behavioral factors, not PhA alone.

### 4.3. CLAD

No significant differences were observed in the BOS stage between patients with different habitual PhA levels, regardless of sex, underlying pulmonary disease, or secondary PH. This suggests that PhA alone may not directly influence BOS progression or graft deterioration. The relationship is likely multifactorial—affected by disease duration, time since transplantation, underlying pathology, and immunosuppressive therapy—which all could mask potential benefits of PhA. However, it should be noted that BOS reflects only one phenotype of CLAD and does not capture less common restrictive forms, which require body plethysmography for proper assessment. BOS was therefore used as a spirometry-based measure allowing uniform comparisons between patients.

Supporting this notion, Pehlivan et al. [45,46] showed that 8–12 weeks of hybrid pulmonary prehabilitation improved dyspnea, 6MWT performance (up to 100 m), and QoL in LTx candidates, but not FEV_1_. Thus, PhA enhanced functional capacity and psychological well-being but did not necessarily modify spirometry or BOS progression.

### 4.4. LOS

Consistent with previous report, pre-transplant habitual PhA—whether none, recreational, or intense—did not significantly influence the LOS after LTx, regardless of sex, pulmonary disease type, or secondary PH. This suggests that postoperative recovery may be mainly determined by acute clinical factors such as perioperative complications, graft function, or immunosuppressive therapy, rather than baseline activity alone.

Across LTx populations, evidence linking prehabilitation to LOS remains inconsistent. Li et al. [26] reported that pre-transplant rehabilitation preserved exercise capacity, and higher pre-transplant 6MWD correlated with shorter LOS. In contrast, Messier et al. [47] and Wickerson et al. [48] found no association between functional performance and postoperative outcomes—mechanical ventilation time, ICU, and overall stay. Similar discrepancies are noted in kidney and liver transplant cohorts: while McAdams-DeMarco et al. [49] and Morkane et al. [50] observed reduced hospitalization (5 vs. 10 days, 13 vs. 30 days) following structured prehabilitation, Al-Judabi et al. [51] found only a modest, nonsignificant trend. These findings and ours suggest that LOS duration depends more on exercise program characteristics (intensity, supervision, duration) and individual clinical characteristics than on baseline PhA alone.

### 4.5. SLTx vs. DLTx

In our study, comparisons between physically active single-lung recipients and inactive double-lung recipients, as well as between groups overall, showed no significant differences in post-transplant outcomes. Functional capacity, BOS stage, and LOS were comparable, indicating that neither transplant type nor habitual PhA substantially influenced early recovery. A moderate effect size in qualitative 6MWT may indicate a compensatory effect of DLTx counterbalancing the potential advantage of previous exercise habits seen in SLTx recipients, though this observation requires cautious interpretation given the multifactorial exploratory analysis and limited sample size.

The literature comparing SLTx and DLTx outcomes remains inconclusive. Several studies [52,53,54] reported superior pulmonary function (FEV_1_ FVC) and exercise capacity (6MWT) in DLTx recipients, though Pochettino et al. [52] found no difference in BOS incidence. In contrast, Neurohr et al. [53] and Gerase et al. [54] observed improved BOS-free and overall survival after DLTx. Other investigations, however, showed comparable recovery and LOS between groups [55,56], suggesting that early outcomes may depend more on perioperative and immunological factors than graft number alone. Overall, these findings imply that although DLTx provides superior ventilatory reserve, it does not translate into shorter hospitalization or faster recovery and may overshadow any beneficial effect of habitual PhA.

### 4.6. Summary

Long-term habitual PhA before LTx shows limited direct effects on postoperative outcomes, which are multifactorial and cannot be explained by PhA alone. However, regular activity remains a key modifiable component of transplant care and likely contributes to physiological resilience and greater regenerative potential. Assessing life-course PhA into clinical evaluation may help identify patients benefiting most from targeted interventions. Incorporating individualized, supervised, and hybrid prehabilitation strategies offers a practical path toward optimizing outcomes in transplant medicine.

Several limitations should be acknowledged. The relatively small and heterogeneous sample likely underpowered statistical comparisons, and the absence of a control group restricted causal inference. Reliance on the long-term retrospective self-reported PhA may have introduced recall bias, especially given the long time period assessed, and could have led to misclassification. Although the questionnaire was administered with structured guidance, this limitation cannot be fully eliminated.

Key psychological factors (e.g., motivation, anxiety), prior exposures (e.g., smoking, occupational, environmental), immunosuppressive therapy, and time since transplantation were not included. The absence of multivariable modeling further limits adjustment for potential confounders. The lack of readmission and long-term mortality data also limited the assessment of the broader prognostic relevance of habitual PhA. In addition, as the study included only patients who were alive at the time of the assessment and able to complete the questionnaire, a potential survivor bias cannot be excluded.

Future research should employ larger, multicenter, longitudinal designs to better clarify the long-term effects of habitual PhA.

## 5. Conclusions

Taken together, our results indicate that long-term habitual PhA before LTx does not significantly affect early post-transplant outcomes, including functional capacity, CLAD, or LOS. Post-transplant recovery appears multifactorial—shaped not only by pre-existing fitness but also by disease duration, comorbidities, treatment, psychological resilience, and overall clinical condition. Lifestyle and environmental influences (smoking history, occupational or chemical exposures, nutrition) also likely contribute and deserve further investigation.

Existing evidence reinforces this complexity. Barriers such as fatigue, comorbidities, and low motivation frequently limit adherence to exercise, whereas habit formation, goal setting, and professional supervision by multidisciplinary teams facilitate sustained PhA [21,57]. Maintaining physical fitness in transplant candidates is further challenged by muscle wasting, treatment side effects, and chronic conditions [58]. Nevertheless, structured or remotely supervised exercise programs have proven effective in preserving or improving pre-transplant functional status [30,44], underscoring the importance of integrated prehabilitation strategies.

This study offers novel insights into the long-term influence of habitual PhA before LTx, integrating functional (6MWT), clinical (CLAD), and hospitalization (LOS) outcomes and uniquely incorporating BOS stage as an endpoint. Finally, the innovative use of a life-course PhA instrument (HAPAQ) represents a key strength of this study, as it uniquely captures long-term behavioral patterns and provides a more robust estimate of pre-transplant physiological reserve, offering a solid foundation for future research.

## Figures and Tables

**Table 1:** Results of two-way analyses of variance testing the relationships between sport groups and sex, type of pulmonary disease, and the presence of secondary PH with the 6MWT result.

Tested Effect	MS	F	df	*p*	η^2^
Sport groups	1498.16	0.09	2; 85	0.911	<0.01
Sex	35,864.58	2.24	1; 85	0.138	0.02
Sport groups × sex	4155.43	0.26	2; 85	0.772	0.01
Sport groups	2870.47	0.18	2; 85	0.832	<0.01
Obstruction/Restriction	110.66	0.01	1; 85	0.933	<0.01
Sport groups × Obstruction/Restriction	3224.59	0.21	2; 85	0.814	0.01
Sport groups	4007.80	0.26	2; 85	0.773	0.01
Secondary PH	1077.40	0.07	1; 85	0.793	<0.01
Sport groups × Secondary PH	1869.41	1.20	2; 85	0.306	0.03

Note. df—degrees of freedom; F—test statistic value; MS—mean square; *p*—statistical significance; η^2^—effect size index. The dependent variable in each tested model was the 6MWT result [m].

**Table 2:** Comparison of patients differentiated by PhA levels in terms of the qualitative outcome of the 6MWT, overall and within groups stratified by sex, obstructive/restrictive disease, and the presence of secondary PH.

		No Sport		Recreational Sport		Intensive Sport				
STUDY GROUP	6MWT Result	N	%	N	%	N	%	χ^2^(4)	*p*	Vc
Overall	Below norm	5	23.8%	10	26.3%	7	18.4%	2.82	0.589	0.12
	Within norm	11	52.4%	24	63.2%	26	68.4%			
	Above norm	5	23.8%	4	10.5%	5	13.2%			
Women	Below norm	3	25.0%	5	19.2%	6	19.4%	1.41	0.843	0.10
	Within norm	6	50.0%	17	65.4%	21	67.7%			
	Above norm	3	25.0%	4	15.4%	4	12.9%			
Men	Below norm	2	22.2%	5	41.7%	1	14.3%	4.01	0.404	0.27
	Within norm	5	55.6%	7	58.3%	5	71.4%			
	Above norm	2	22.2%	0	0.0%	1	14.3%			
Obstruction	Below norm	3	30.0%	7	36.8%	0	0.0%	5.89	0.208	0.28
	Within norm	4	40.0%	10	52.6%	6	66.7%			
	Above norm	3	30.0%	2	10.5%	3	33.3%			
Restriction	Below norm	2	20.0%	2	12.5%	5	20.8%	2.66	0.633	0.16
	Within norm	6	60.0%	12	75.0%	18	75.0%			
	Above norm	2	20.0%	2	12.5%	1	4.2%			
Secondary PH	Below norm	2	18.2%	2	12.5%	3	17.6%	0.717	0.949	0.09
	Within norm	8	72.7%	11	68.8%	12	70.6%			
	Above norm	1	9.1%	3	18.8%	2	11.8%			
No secondary PH	Below norm	3	33.3%	7	38.9%	2	13.3%	10.25	**0.036**	0.35
	Within norm	2	22.2%	10	55.6%	11	73.3%			
	Above norm	4	44.4%	1	5.6%	2	13.3%			

Note. N—number of observations; *p*—statistical significance; Vc—effect size index; χ^2^—chi-square test result.

**Table 3:** Comparison of patients differentiated by PhA levels in terms of the BOS stage, overall and within groups stratified by sex, obstructive/restrictive disease, and the secondary PH.

		BOS Stage					
		Mean Rank	Me	IQR	H(2)	*p*	η^2^
Overall	No sport (n = 20)	39.10	0.00	0.00–0.00	2.90	0.235	0.01
	Recreational sport (n = 34)	46.32	0.00	0.00–1.00			
	Intensive sport (n = 36)	48.28	0.00	0.00–1.00			
Women	No sport (n = 12)	26.38	0.00	0.00–0.00	2.96	0.227	0.01
	Recreational sport (n = 24)	32.64	0.00	0.00–1.00			
	Intensive sport (n = 30)	34.85	0.00	0.00–1.00			
Men	No sport (n = 8)	12.81	0.00	0.00–0.00	0.367	0.832	<0.01
	Recreational sport (n = 12)	14.17	0.00	0.00–0.75			
	Intensive sport (n = 6)	13.08	0.00	0.00–0.50			
Obstruction	No sport (n = 10)	17.00	0.00	0.00–0.00	1.62	0.445	<0.01
	Recreational sport (n = 18)	18.11	0.00	0.00–0.00			
	Intensive sport (n = 8)	21.25	0.00	0.00–1.00			
Restriction	No sport (n = 9)	18.67	0.00	0.00–0.00	3.01	0.222	0.01
	Recreational sport (n = 13)	26.50	0.00	0.00–1.00			
	Intensive sport (n = 23)	22.72	0.00	0.00–1.00			
Secondary PH	No sport (n = 10)	17.30	0.00	0.00–0.00	1.95	0.378	<0.01
	Recreational sport (n = 14)	22.36	0.00	0.00–1.00			
	Intensive sport (n = 16)	20.88	0.00	0.00–0.75			
No secondary PH	No sport (n = 9)	18.50	0.00	0.00–0.00	1.26	0.531	<0.01
	Recreational sport (n = 16)	19.91	0.00	0.00–0.00			
	Intensive sport (n = 15)	22.33	0.00	0.00–1.00			

Note. H—test statistic value; IQR—interquartile range; Me—median; *p*—statistical significance; η^2^—effect size index.

**Table 4:** Results of two-way analysis of variance testing the associations between sport groups and sex, obstructive/restrictive disease, presence of secondary PH, and LOS.

Tested Effect	MS	F	df	*p*	η^2^
Sport groups	325.51	0.40	2; 85	0.673	0.01
Sex	136.24	1.67	1; 85	0.200	0.02
Sport groups × Sex	417.19	0.51	2; 85	0.603	0.01
Sport groups	324.71	0.46	2; 85	0.635	0.01
Obstruction/Restriction	386.28	0.54	1; 85	0.463	0.01
Sport groups × Obstruction/Restriction	643.51	0.90	2; 85	0.409	0.02
Sport groups	364.31	0.50	2; 85	0.608	0.01
Secondary PH	4.26	0.01	1; 85	0.939	<0.01
Sport groups × Secondary PH	18.98	0.03	2; 85	0.974	<0.01

Note. df—degrees of freedom; F—test statistic value; MS—mean square; *p*—statistical significance; η^2^—effect size index.

**Table 5:** Comparison of physically active patients after SLTx with physically inactive patients after DLTx in terms of the 6MWT result, BOS stage, and LOS.

	Physically Active Patients After SLTx	Physically Inactive Patients After DLTx			
	M ± SD/n (%)/ Me (IQR)	M ± SD/n (%)/ Me (IQR)	t/Z/χ^2^	*p*	Cohen’s d/η^2^/Vc
6MWT—quantitative	439.50 ± 140.72	490.18 ± 132.95	1.03	0.312	0.37
6MWT—qualitative			4.09	0.129	0.36
Below norm	3 (21.4%)	4 (23.5%)			
Within norm	11 (78.6%)	9 (52.9%)			
Above norm	0 (0.0%)	4 (23.5%)			
BOS stage	0.00 (0.00–0.25)	0.00 (0.00–0.00)	1.13	0.260	0.04
LOS	45.21 ± 22.75	43.71 ± 21.20	0.19	0.850	0.07

Note. IQR—interquartile range; M—mean; Me—median; n—group size; *p*—statistical significance; SD—standard deviation; t/Z/χ^2^—test statistic; Cohen’s d/η^2^/Vc—effect size indices. For the 6MWT (quantitative) and LOS, Student’s *t*-test for independent samples was reported along with Cohen’s d effect size. For the BOS stage, the Mann–Whitney test was reported with η^2^ as the effect size. For the 6MWT (qualitative), the chi-square test of independence was reported with Vc as the effect size.

## Data Availability

The data generated and analyzed within this study are not publicly available due to their confidential nature.

## Abbreviations

The following abbreviations are used in this manuscript:

6MWT	6 min walk test
ANOVA	analysis of variance (two-way between-subjects)
BOS	bronchiolitis obliterans syndrome
CLAD	chronic lung allograft dysfunction
COPD	chronic obstructive pulmonary disease
DLTx	double lung transplantation
FEV_1_	forced expiratory volume in one second
FVC	forced vital capacity
HAPAQ	Historical Adulthood Physical Activity Questionnaire
IPAQ	International Physical Activity Questionnaire
LOS	length of hospital stay
LTx	lung transplantation
MLTPAQ	Minnesota Leisure Time Physical Activity Questionnaire
PhA	physical activity
PASE	Physical Activity Scale for the Elderly
PH	pulmonary hypertension
QoL	quality of life
SLTx	single lung transplantation
TOAQ	Typical Adult Physical Activity Questionnaire
